# Supplementary material for: The GIAB genomic stratifications resource for human reference genomes
Source: Nat Commun. 2024 Oct 19;15:9029. doi: 10.1038/s41467-024-53260-y (PMC11489684; doi:10.1038/s41467-024-53260-y)
Supplement: Supplementary file 1 — Supplementary Information [file 41467_2024_53260_MOESM1_ESM.pdf]

Supplementary Information

Supplementary Note 1. Stratification overview.
Supplementary Figure 1. Coverage of all low-mappability (difficult to map) regions.
Supplementary Figure 2. Coverage of regions with high (>85%) GC content.
Supplementary Figure 3. Comparison of variants in syntenic and non-syntenic regions.
Supplementary Figure 4. The distribution of genomic distance variants along each chromosome.
Supplementary Table 1. Data sources used for the stratification benchmark comparisons.
Supplementary Table 2. Summary of data requirements for stratification categories.

Supplementary Note 1

Stratification overview

In this section, we provide an overview of existing genomic stratifications for GRCh37/38 including regions with high/low GC content, low complexity, low mappability, and segmental duplications, in addition to regions with patterns of local ancestry, chromosome XY specific regions and other difficult genomic regions.

Gene coding regions

Coding sequences (CDS) are the regions of the genome that code for proteins. These are usually targeted for many clinical tests. Since many diseases are caused by variants in protein sequences, being able to find variants in their corresponding genome sequences is important in clinical applications[6]. For GRCh37 and GRCh38, the coding regions were defined using the RefSeq annotations dataset[10].

Low mappability regions

To identify whether reads of a given length will align uniquely to a region of the genome, the reference mappability metric is utilized. One method to identify such regions is part of the GEnome Multitool (GEM)[44]. This tool queries the genome for sequences of a given length that align other places; these alignments may be allowed some upper limit of mismatches and gaps, corresponding respectively to SNVs and INDELs. For GRCh37/38, current stratifications include two levels of mappability stringency. The "low stringency" set includes all regions 100bp long that align somewhere else within the genome with no more than two SNVs and one INDEL. The "high stringency" set includes all regions 250bp long that perfectly align other places within the genome without any SNVs or INDELs. The lengths for these mappability stratifications roughly correspond to those commonly provided by short read sequencing technologies.

GC Content

The regions where the fraction of G and C bases is high or low are simply defined as high and low GC-content regions. Such definition is important as different sequencing technologies can produce distinct error profiles in GC-rich and AT-rich regions[16]. For example, GC-rich and AT-rich regions tend to have reduced coverage for many technologies[47]. GC-rich and AT-rich regions also can have reduced precision and recall in variant calling[48]. For GRCh37/38, these regions have been determined using the seqtk algorithm[49]. We identify regions with specific GC percentage in 5% increments from 15-85%.

Segmental Duplications

DNA sequences longer than 1kbp with high sequence identity (typically >90%) that are repeated in a genome are called segmental duplications (segdups)[27]. Segmental duplications play important roles in genome evolution and many diseases[50]. The GIAB stratifications define segdups based on two data sources. The first is defined from genomicSuperDups[51] hosted at the UCSC genome browser. This dataset defines the "canonical" segmentally duplicated regions, *e.g.*, regions >1kbp with 90% similarity without other repeat regions such as LINEs or SINEs. These stratification regions were identified by merging all regions from either source database and removing the Pseudoautosomal regions (PAR) from the X and Y chromosomes because these were incorrectly determined to be segmental duplications. The stratifications include two sets of BED files for each source: one with all regions and one with regions >10kb.

Regions with low complexity sequences

Another genomic stratification indicates regions with low complexity sequences. We define "low complexity" to include perfect and imperfect homopolymers, tandem repeats, and satellites. A homopolymer is defined as a sequence of consecutive identical bases. Tandem repeats refer to motifs that repeat up to a given length. Depending on the motif length they can either be short tandem repeats (STRs, also known as microsatellites) which have a motif size of 2-6 bases or variable number tandem repeats (VNTRs) which can have longer motifs. Satellites have repeating motifs like tandem repeats, but the total length of the repeat is generally much longer and motifs are more complex. The longest satellites occur in centromeric regions.

To construct the low complexity stratifications we utilized several sources. First, we queried the RepeatMasker database[52] and filtered for "Low Complexity", "Simple Repeat", and "Satellite" classes. Next, we queried Tandem Repeat Finder (TRF)[53] to obtain tandem repeats. Finally, we found homopolymers as well as exact repeating sequences with motif size up to 4 bases using a custom script, because the other resources missed some shorter homopolymers and tandem repeats associated with sequencing errors. We identified both perfect repeats (*i.e.*, the same base or bases repeated identically) and imperfect repeats (*i.e.*, repeated sequences with small differences from the repeat motif). To generate the final bed files, we merged these data sources using bedtools (since many regions are expected to overlap) and binned each bed file according to length, since longer repeated regions are expected to be more difficult from a sequencing and variant-calling perspective.

Chromosome XY specific regions

Some regions specific to sex chromosomes need to be defined as stratifications due to their importance and distinct evolution of repeats. The human X and Y chromosomes share two pseudoautosomal regions (PARs including PAR1 and PAR2) at the ends of the chromosomes that continue to undergo homologous X-Y recombination[13]. In addition to these, the X-chromosome-transposed region (XTR, sometimes also called PAR3) was duplicated from the X to the Y chromosome in humans after human-chimpanzee divergence and is known to be a recombination hotspot resulting in deletions and inversions[54]. Chromosome Y also includes the ampliconic gene families with highly homologous genes. A typical human Y chromosome harbors 16 single-copy protein-coding X-degenerate genes, with housekeeping functions and homologs on the X chromosome; and 9 protein-coding ampliconic gene families, which have expanded specifically on the Y chromosome[13,54]. Individual stratification BED files were created for PAR, XTR, and ampliconic regions.

Patterns of local ancestry in the reference genome

It can be important to know the ancestry of the individuals' haplotypes that are part of a reference genome because abrupt changes in ancestry of reference regions can cause

challenges with linkage disequilibrium (LD) when aligning reads to the reference[18], and because regions of different ancestry can impact the number of variants called[55]. Local ancestry describes the origin of a chromosomal segment in terms of geographic and regional population. The majority continental super-population affiliation of 1000 Genomes Project samples that most closely match GRCh38 intervals were reported[56]. This was done for African ancestry (AFR), American ancestry (AMR), East-Asian ancestry (EAS), European ancestry (EUR), South-Asian ancestry (SAS). We also report intervals of putative Neanderthal-introgressed origin, based on inferred patterns of identity-by-descent with the Vindija Neanderthal genome [12,18]. This stratification is currently provided only for GRCh38.

Other difficult genomic regions

Some regions of the genome are difficult to analyze due to high degrees of polymorphism or limitations in the reference. The list of "Other difficult genomic regions" includes contigs in the reference assembly that are smaller than 500kbp and all gaps in the reference assembly. This category also includes the Major Histocompatibility Complex (MHC) on chromosome 6[57], the variable/diversity/joining (VDJ) regions on 2, 14 and 22, and the Killer-cell immunoglobulin-like receptor (KIR) region[17]. These three regions are all highly polymorphic and underpin key immunological functions: the MHC region contains the Human Leukocyte Antigen (HLA) genes which determine "donor matches", the VDJ regions are randomly recombined to produce the T and B cell receptors, and the KIR region codes for one of the key effector receptors on natural killer cells. Stratifications for these difficult regions exist for GRCh37 and GRCh38.

Functional, technically difficult to sequence

Several coding regions of the genome present with one of several difficulties. First, some transcription start sites or first exons in the human genome tend to have poor coverage, making accurate sequencing difficult[47]. The stratification defined for these regions include the first 1000 promoters with the lowest coverage by Illumina[47]. Second, some genes are known to be duplicated in most individuals relative to the existing references, which leads to mapping difficulties; this includes KMT2C in both GRCh37 and GRCh38. Third, some genes in the reference are falsely duplicated, which similarly leads to mapping issues. Genes in this category include MRC1 and part of CNR2 (GRCh37) as well as CBS, CRYAA, KCNE1, and H19 (GRCh38).

Genome specific

Seven GIAB samples including HG001, HG002, HG003, HG004, HG005, HG006, and HG007 are studied to identify genome-specific difficult regions. These regions cover putative compound heterozygous variants, multiple variants within 50bp of each other, and potential structural variations and copy number variations. These stratifications could be used with benchmarking tools like hap.py to stratify variant calls in terms of true positive, false positive, and false negative.

Supplementary Figures

A.

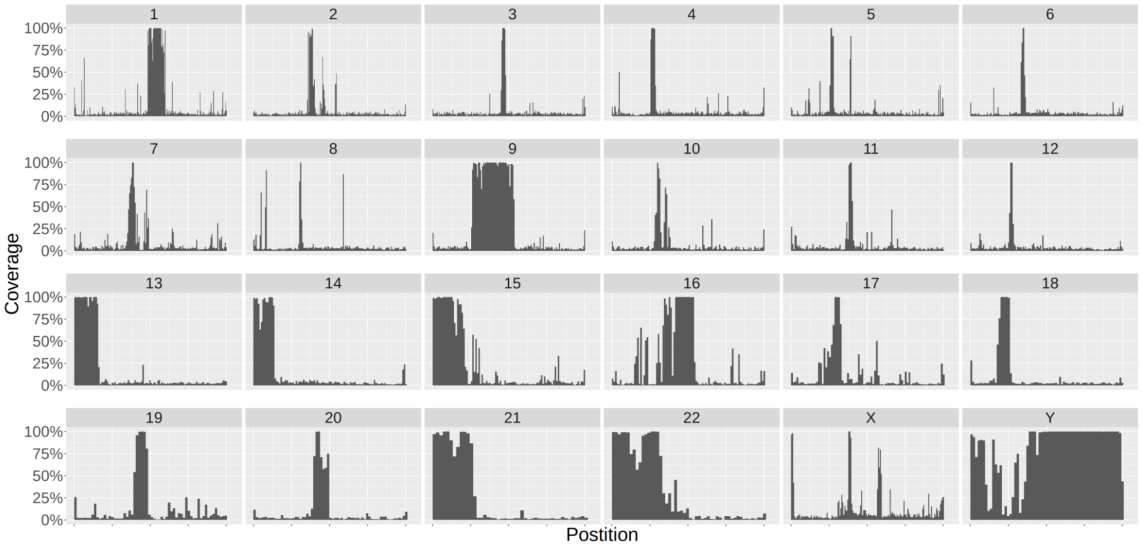

B.

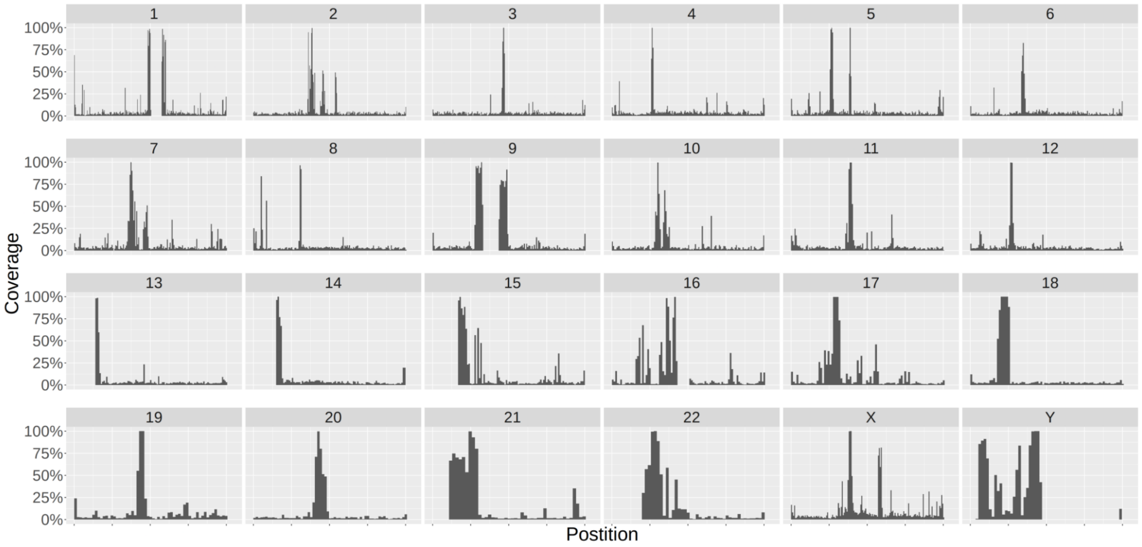

Supplementary Figure 1: Coverage of all low-mappability (difficult to map) regions for a) CHM13 and b) GRCh38. Coverage was calculated in 1Mbp windows as the fraction within the "lowmappabilityall" stratification bed file.

A.

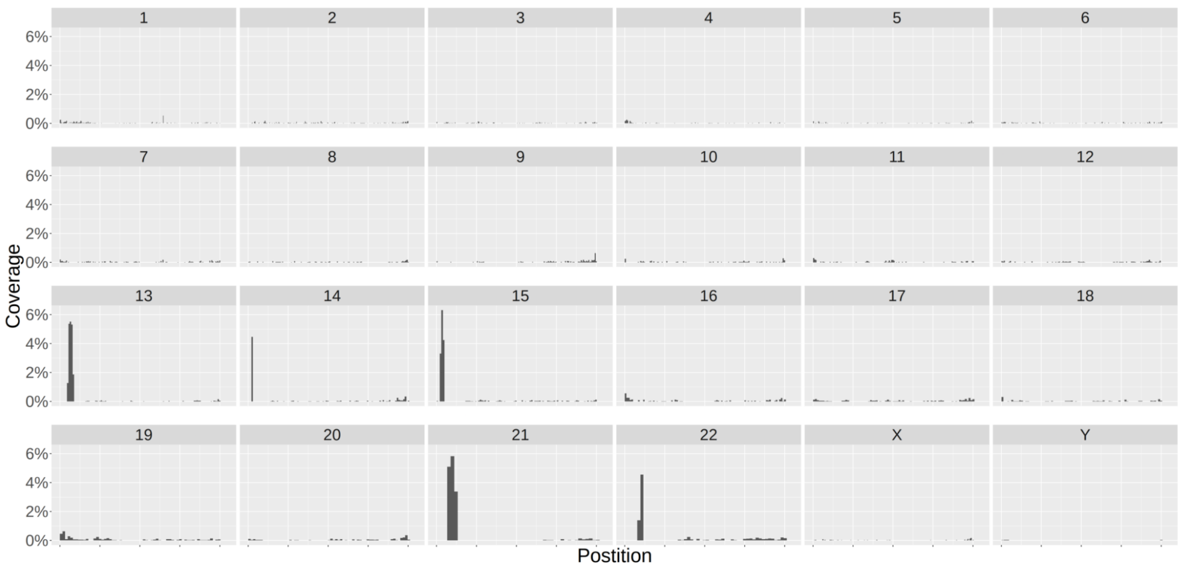

B.

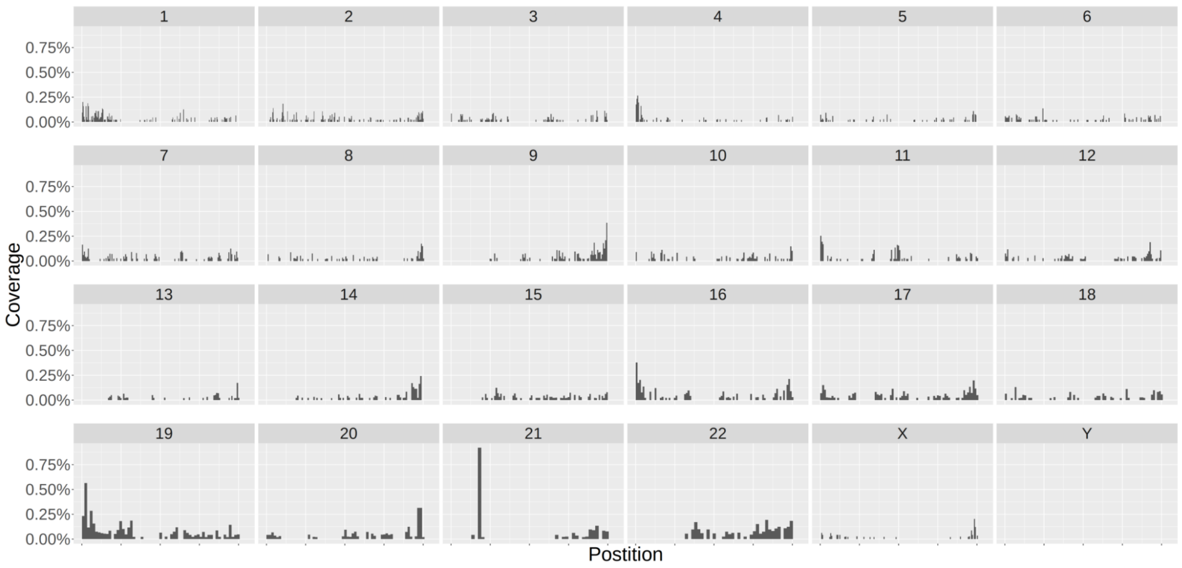

Supplementary Figure 2: Coverage of regions with high (>85%) GC content for a) CHM13 and b) GRCh38. Coverage was calculated in 1Mbp windows as the fraction within the “gc85_slop50” stratification bed file.

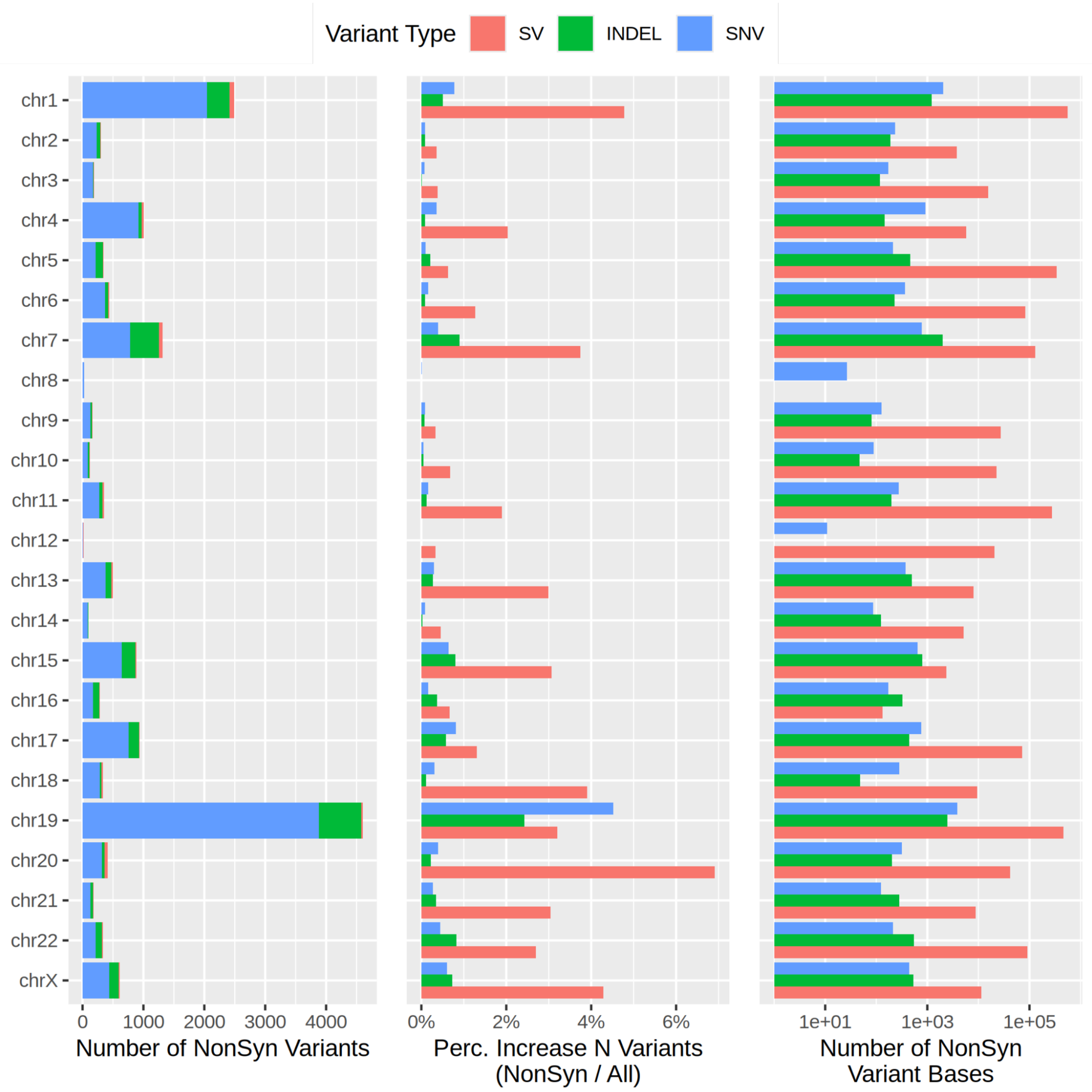

Supplementary Figure 3: Comparison of variants in syntenic and non-syntenic regions. SV = structural variant (variant 50 bp or longer).

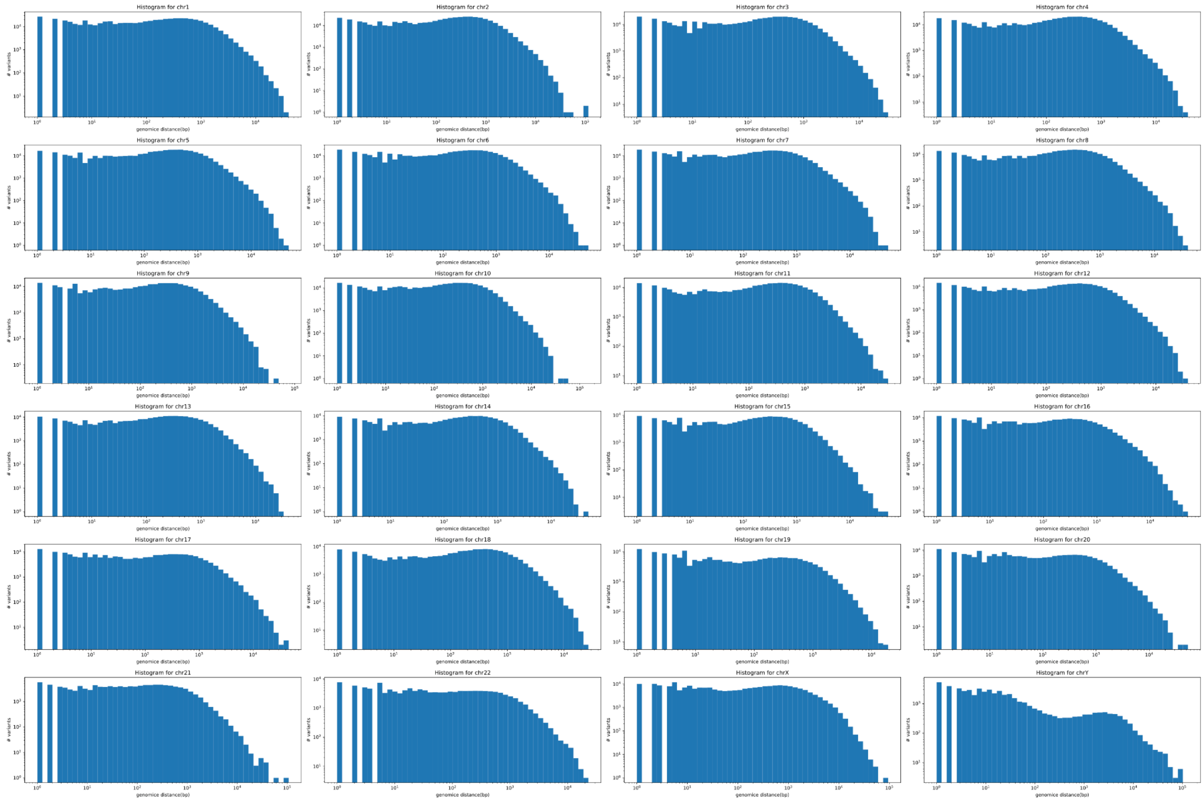

Supplementary Figure 4: The distribution of genomic distance variants along each chromosome. We should note that a portion of the genome is unknown (existing as Ns in the reference file) and is excluded in this figure. The reason is that no variant can be found in these regions.

Supplementary Tables

Supplementary Table 1: Data sources used for the stratification benchmark comparisons

Description	Analyses	Url
GRCh38 (non-ONT comparison) fasta	Figure 4a	https://giab-data.s3.amazonaws.com/giab-test-data/GRCh38_GIABv3_no_alt_analysis_set_maskedGRC_decoys_MAP2K3_KMT2C_KCNJ18.fasta.gz
GRCh38 (ONT comparison) fasta<br><br>NOTE: this was required since the corresponding callsets used ambiguous bases	Figure 4c	https://ftp.ncbi.nlm.nih.gov/genomes/all/GCA/000/001/405/GCA_000001405.15_GRCh38/seqs_for_alignment_pipelines.ucsc_ids/GCA_000001405.15_GRCh38_no_alt_analysis_set.fna.gz
GRCh37 fasta	Figure 4a	https://ftp-trace.ncbi.nlm.nih.gov/ReferenceSamples/giab/release/references/GRCh37/hs37d5.fa.gz
CHM13 fasta	Figure 4a-b	https://s3-us-west-2.amazonaws.com/human-pangenomics/T2T/CHM13/assemblies/analysis_set/chm13v2.0.fa.gz
CHM13 non-syntenic bed file (relative to GRCh38)	Figure 4b	https://hgdownload.soe.ucsc.edu/gbdb/hs1/hgUnique/hgUnique.hg38.bb
HiFi Callset (GRCh38)	Figure 4a-b	https://ftp-trace.ncbi.nlm.nih.gov/ReferenceSamples/giab/data/AshkenazimTrio/analysis/PacBio_HiFi-Revio_20231031/pacbio-wgs-wdl_germline_20231031/HG002.GRCh38.deepvariant.phased.vcf.gz
HiFi Callset (GRCh37)	Figure 4a-b	https://ftp-trace.ncbi.nlm.nih.gov/ReferenceSamples/giab/data/AshkenazimTrio/analysis/PacBio_HiFi-Revio_20231031/pacbio-wgs-wdl_germline_20231031/HG002.GRCh37.deepvariant.phased.vcf.gz
HiFi Callset (CHM13)	Figure 4a-b	https://ftp-trace.ncbi.nlm.nih.gov/ReferenceSamples/giab/data/AshkenazimTrio/analysis/PacBio_HiFi-Revio_20231031/pacbio-wgs-wdl_germline_20231031/HG002.CHM13.deepvariant.phased.vcf.gz
Stratifications (for GRCh37,	Figure 4	https://ftp-

GRCh38, and CHM13)		trace.ncbi.nlm.nih.gov/ReferenceSamples/giab/release/genome-stratifications/v3.3/genome-stratifications-GRCh38@all.tar.gz
ONT guppy4+clair1 callset	Figure 4c	http://www.bio8.cs.hku.hk/clair3/analysis_result/ont_guppy4/2_coverage_subsampling/clair/hg003_40x_clair_filter_q748.vcf.gz
ONT guppy 5+clair3 callset	Figure 4c	http://www.bio8.cs.hku.hk/clair3/analysis_result/ont_guppy5/2_coverage_subsampling/clair3/hg003_40x_clair3.vcf.gz
Q100 Assembly-based HG002 benchmark (for GRCh37, GRCh38, CHM13, we used the *.vcf.gz and *_smvar.benchmark.bed files)	Figure 4a-b	https://ftp-trace.ncbi.nlm.nih.gov/ReferenceSamples/giab/data/AshkenazimTrio/analysis/NIST_HG002_DraftBenchmark_defrabbV0.015-20240215/
GIAB v4.2.1 HG003 benchmark (vcf and bed file)	Figure 4c	https://ftp-trace.ncbi.nlm.nih.gov/ReferenceSamples/giab/release/AshkenazimTrio/HG003_NA24149_father/NISTv4.2.1/GRCh38/
HG002 Illumina callset	Figure 5f-h	https://storage.googleapis.com/brain-genomics-public/research/sequencing/grch38/vcf/hiseqx/wgs_pcr_free/40x/HG002.hiseqx.pcr-free.40x.deepvariant-v1.0.grch38.vcf.gz

Supplementary Table 2: Summary of data requirements for stratification categories. Those marked “none” only require the reference fasta (which is required in all cases). Full provenance information can be found in the READMEs for each reference/stratification category on the FTP site: https://ftp-trace.ncbi.nlm.nih.gov/ReferenceSamples/giab/release/genome-stratifications/v3.5

**Category**	**Required Data**
Functional	Requires a GFF file with CDS regions from RefSeq
GCcontent	none
LowComplexity	● Homopolymers (perfect and imperfect), and 2, 3, and 4-mer repeats can be generated from the fasta only<br>● Satellite regions require censat annotations<br>● Tandem repeats require RepeatMasker and TandemRepeat finder annotations
Mappability	none
OtherDifficult	VDJ regions can be derived from the same GFF file used to create the Functional stratifications. All other files must be manually provided.
SegmentalDuplications	All files require SEDEF annotations
Telomere	none
Union	Requires segmental duplications (see above) and XY XTR and ampliconic regions (see below)
XY	● Pseudoautosomal regions require the coordinates of these regions to be specified in the config<br>● XTR and ampliconic bed files require a tsv with bed coordinates labeled with each region type<br>● The above applies individually to X and Y

47. Ross, M. G. *et al.* Characterizing and measuring bias in sequence data. *Genome Biol.* **14**, R51 (2013).

48. Zhao, S., Agafonov, O., Azab, A., Stokowy, T. & Hovig, E. Accuracy and efficiency of germline variant calling pipelines for human genome data. *Sci. Rep.* **10**, 20222 (2020).

49. Li, H. *Seqtk: Toolkit for Processing Sequences in FASTA/Q Formats*. (Github, 2023).

50. Vollger, M. R. *et al.* Long-read sequence and assembly of segmental duplications. *Nat. Methods* **16**, 88–94 (2019).

51. Bailey, J. A., Yavor, A. M., Massa, H. F., Trask, B. J. & Eichler, E. E. Segmental duplications: organization and impact within the current human genome project assembly. *Genome Res.* **11**, 1005–1017 (2001).

52. RepeatMasker website. http://www.repeatmasker.org (2023).

53. Benson, G. Tandem repeats finder: a program to analyze DNA sequences. *Nucleic Acids Res.* **27**, 573–580 (1999).

54. Cotter, D. J., Brotman, S. M. & Wilson Sayres, M. A. Genetic Diversity on the Human X Chromosome Does Not Support a Strict Pseudoautosomal Boundary. *Genetics* **203**, 485–492 (2016).

55. Goetz, L. H., Uribe-Bruce, L., Quarless, D., Libiger, O. & Schork, N. J. Admixture and clinical phenotypic variation. *Hum. Hered.* **77**, 73–86 (2014).

56. Lowy, E., Fairley, S. & Flicek, P. Variant calling across 505 openly consented samples from four Gambian populations on GRCh38. *Wellcome Open Res.* **6**, 239 (2021).

57. Chin, C.-S. *et al.* A diploid assembly-based benchmark for variants in the major histocompatibility complex. *Nat. Commun.* **11**, 4794 (2020).
